# Simultaneous Bilateral Insufficiency Femoral Fractures After Long-Term Alendronate Treatment: A Lesson Not Yet Learned

**DOI:** 10.7759/cureus.33956

**Published:** 2023-01-19

**Authors:** Dimitrios Rigkos, Ioannis Savvidis, Alexia Bisbinas, Georgios Markopoulos, Ilias Bisbinas

**Affiliations:** 1 1st Trauma and Orthopaedics Department, 424 General Military Training Hospital, Thessaloniki, GRC; 2 Internal Medicine Department, University of Pavia, Pavia, ITA

**Keywords:** atypical fractures, insufficiency femoral fractures, simultaneous, bilateral, bisphosphonates, alendronate

## Abstract

Bisphosphonates have recently been used as a first-line treatment for osteoporosis. However, prolonged bisphosphonate use may be associated with insufficiency and atypical femoral fractures. In this case report, we present a patient with simultaneous bilateral insufficiency femoral fractures after using alendronate for 11 years, which were treated surgically. Our patient also had a history of a previous right femoral atypical fracture eight years before the latest ones, while on 3-year alendronate treatment. To our knowledge, it is the first patient reported with three atypical - insufficiency fractures covering all the anatomical areas of the proximal half of the femur after long-term bisphosphonate treatment.

## Introduction

Bisphosphonates have emerged as one of the most commonly prescribed anti-osteoporotic medications over the past 20 years due to their ability to lower the frequency of femoral neck and vertebral fractures in osteoporotic patients [1]. In 1995, the USA Food and Drug Administration authorized alendronate as the first bisphosphonate for use in osteoporotic fracture prevention [2].

On the other hand, prolonged use of bisphosphonates appears to be related to femoral insufficiency or atypical fractures [3]. Long-term treatment with alendronate can cause severe suppression of bone turnover, increasing the risk of non-vertebral fractures and delaying their recovery [4]. Lately, several case reports have described unilateral or bilateral atypical femoral fractures (AFFs) in patients after bisphosphonate use, but there are only a few references for simultaneous bilateral insufficiency fractures following prolonged alendronate use [5-7]. We describe the surgical management of concurrent bilateral insufficiency femoral fractures after long-term alendronate treatment in a patient with a previous history of an atypical femoral fracture.

## Case presentation

**Figure 1:**
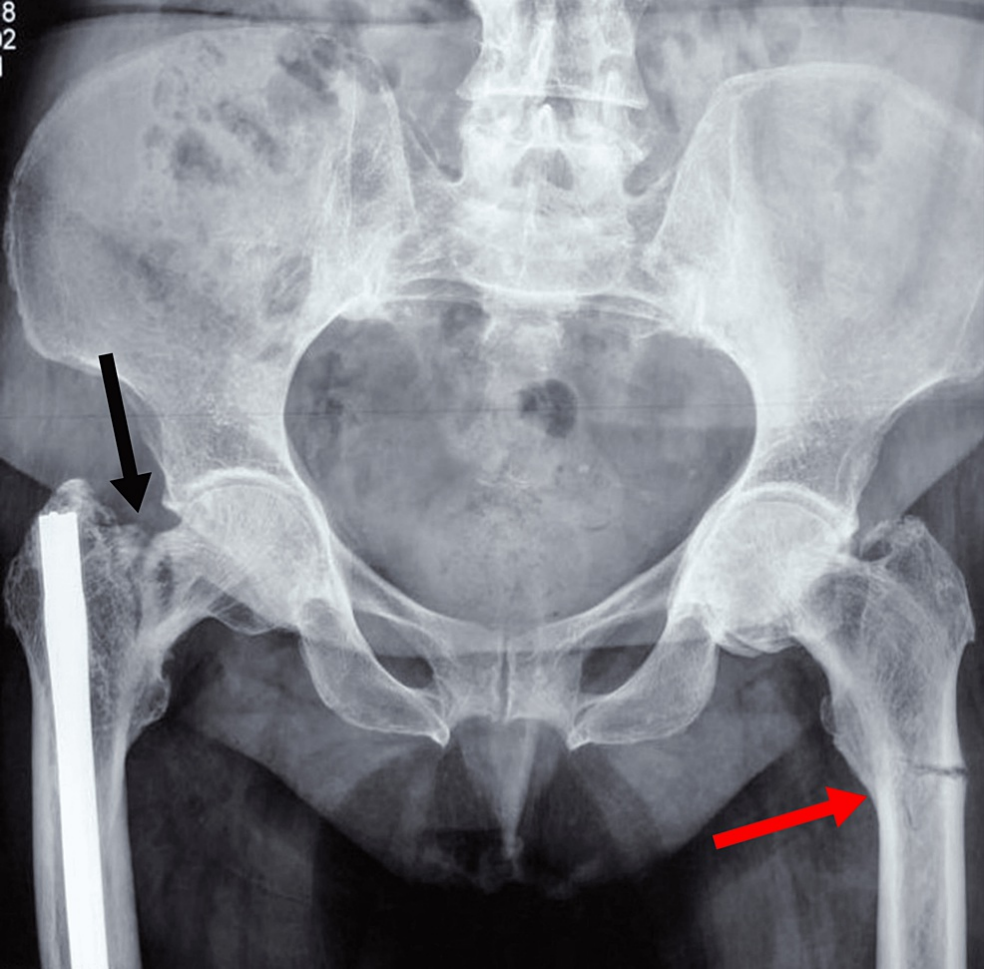
AP pelvis view at the emergency department with simultaneous bilateral femoral fractures Right neck of femur fracture (black arrow) and left subtrochanteric fracture (red arrow) AP: anteroposterior

A 68-year-old female postmenopausal patient was admitted to our hospital complaining of acute bilateral thigh pain and an inability to walk without any trauma history. After clinical and radiographic evaluation, an incomplete right femoral neck fracture and an incomplete left subtrochanteric femoral fracture were noted (Figure 1). The patient reported prodromal signs of bilateral thigh pain and difficulty walking during the last nine months. Although she had quite a few medical consultations elsewhere and image investigations throughout this nine-month period, her symptoms were attributed to lumbar spine pathology.

**Figure 2:**
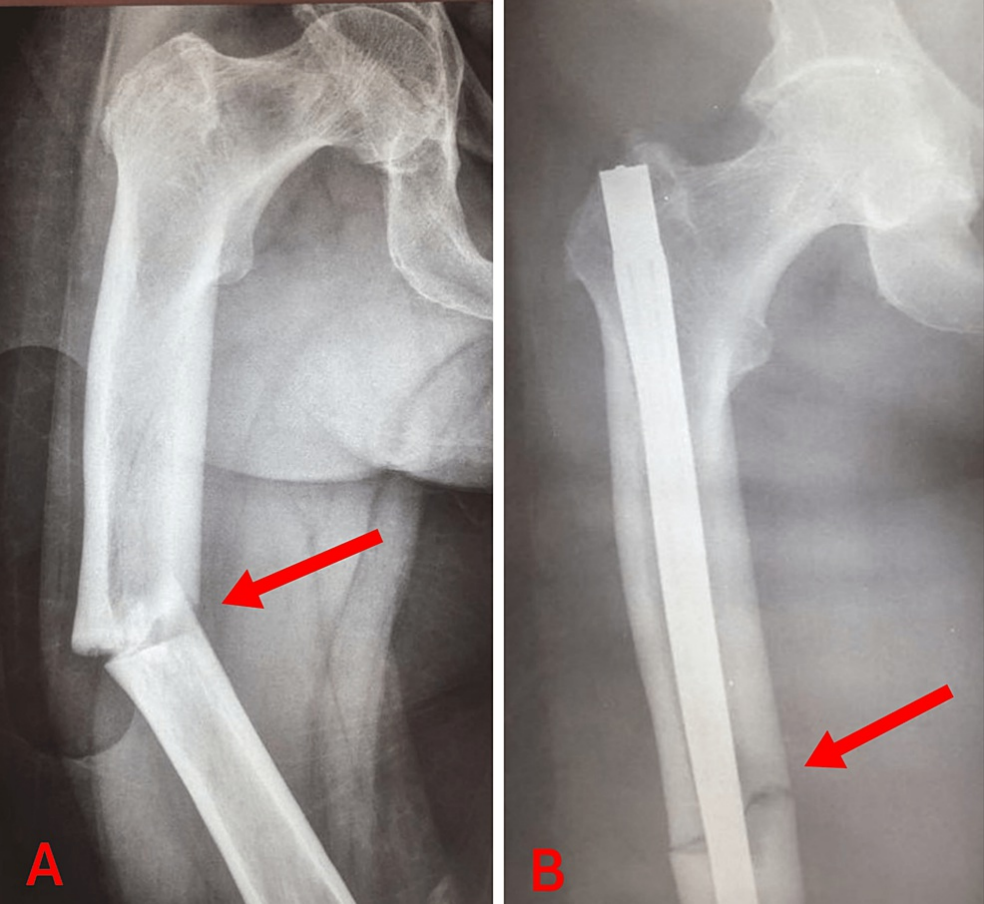
(A) Right atypical femoral diaphysis fracture (red arrow) after three-year use of alendronate; (B) Intramedullary nailing of AFF (red arrow) AFF: atypical femoral fracture

As for her medical history, she was on medication for hypertension, dyslipidemia, type II diabetes mellitus, and arrhythmia. She had also been taking 70 mg of alendronate weekly for the last 11 years for osteoporosis. It should be noted that secondary to osteoporosis, she had developed substantial kyphoscoliosis, which had been treated conservatively. Regarding her surgical history, she had an atypical fracture of her right femoral diaphysis eight years ago after minor trauma, when she had already been on alendronate for three years (Figure 2A). This atypical fracture was surgically fixed with intramedullary (IM) nailing elsewhere (Figure 2B). Her alendronate treatment was not discontinued at that time.

**Figure 3:**
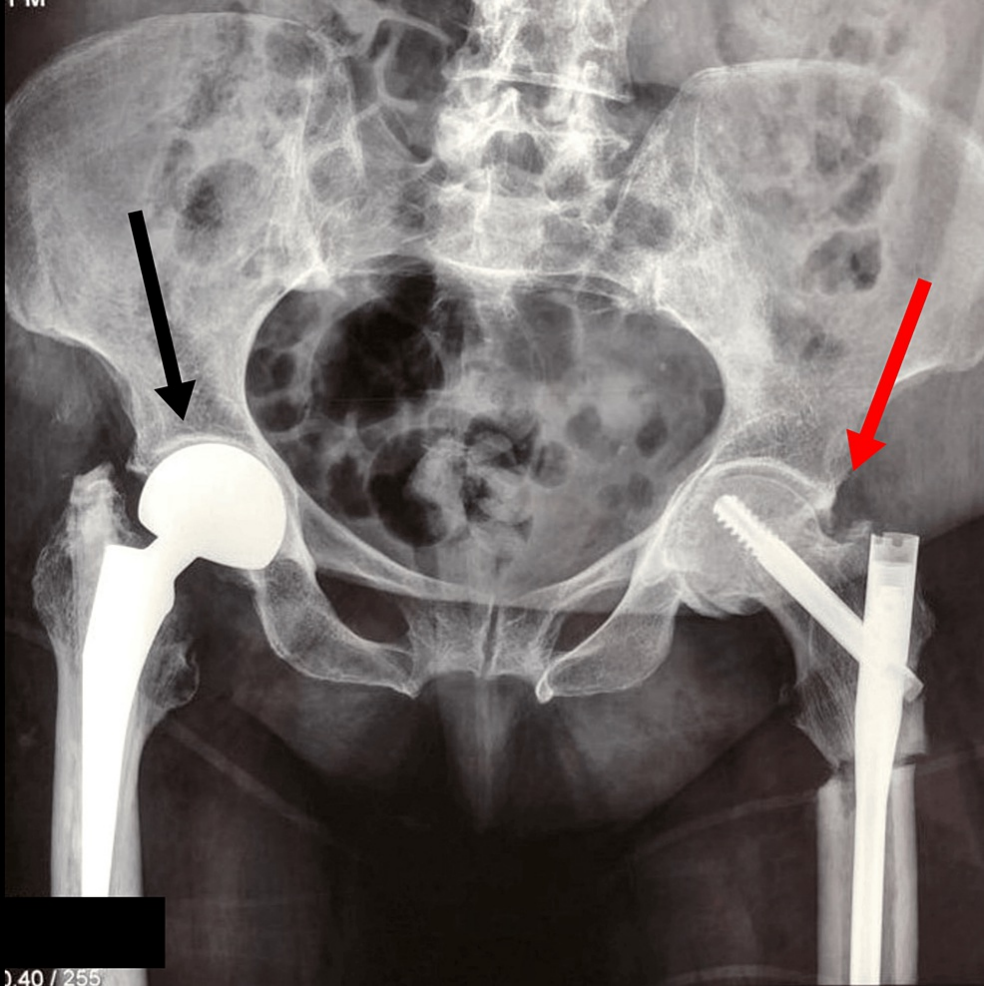
AP pelvis view immediately postoperatively Right hip hemiarthroplasty (black arrow) and left femur intramedullary nailing (red arrow) AP: anteroposterior

After the necessary preoperative management, the patient was treated surgically for both fractures under a single anesthetic. Initially, a right hip hemiarthroplasty was performed after the removal of the IM nail (Figure 3). Subsequently, IM nailing of the left femur with a long Gamma nail was applied (Figure 3). The patient recovered uneventfully, and she was mobilized early postoperatively. Alendronate treatment was discontinued, and she started having denosumab in combination with vitamin D and calcium.

**Figure 4:**
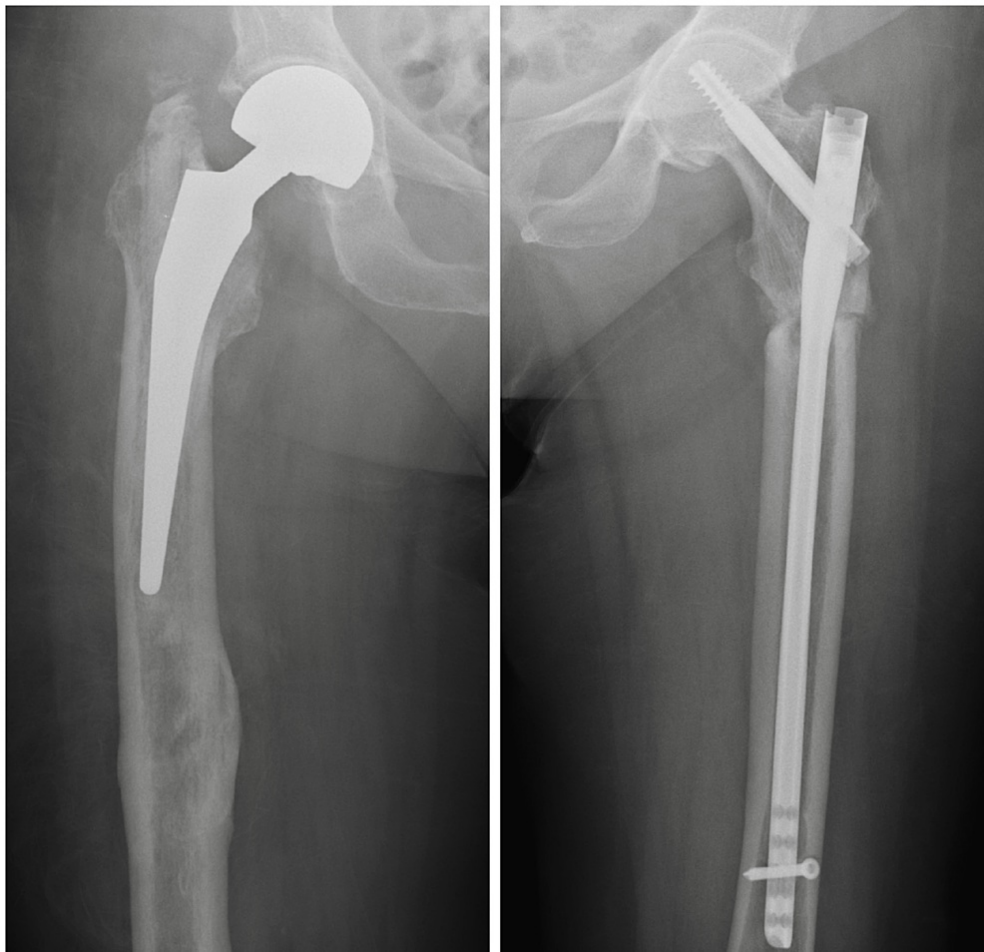
Right hip hemiarthroplasty and left femur intramedullary nailing three months postoperatively

Three months postoperatively, the clinical and radiological outcomes were fine (Figure 4), and the patient was able to mobilize indoors with full weight-bearing using a Zimmer frame (Video 1).

**Video 1:** Patient’s mobilization three months postoperatively

**Figure 5:**
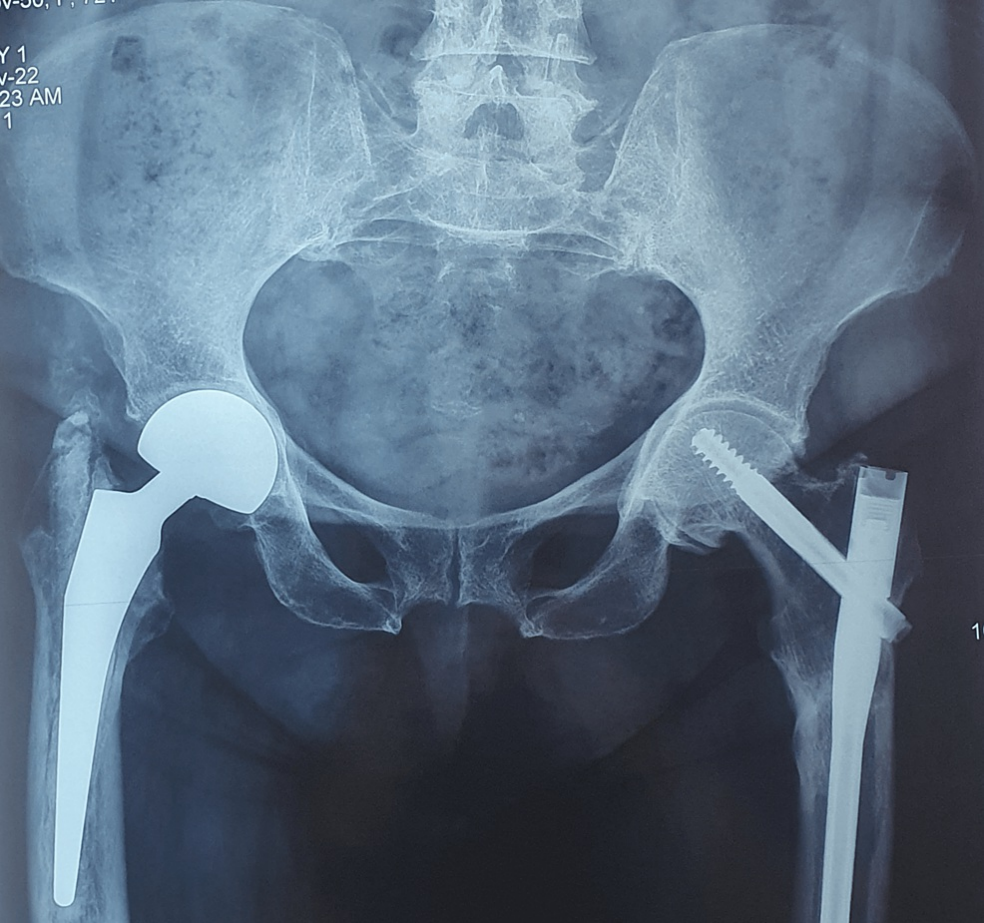
AP pelvis view four years postoperatively AP: anteroposterior

At the final follow-up, four years postoperatively, complete bone healing was confirmed both clinically and radiologically (Figure 5), and the patient was able to walk independently, fully weight-bearing outdoors (Video 2).

**Video 2:** Patient's mobilization four years postoperatively

## Discussion

Bisphosphonates are widely prescribed for the treatment and prevention of osteoporosis [1]. Several authors, however, have already established a strong link between long-term bisphosphonate use and insufficiency femoral fractures [2,3,8,9]. According to the American Society for Bone and Mineral Research (ASBMR), AFFs are stress or insufficiency fractures that occur gradually and show some major and minor features [10]. Major features include association with no or minimal trauma, transverse or short oblique orientation, lack of comminution, and localized periosteal reaction or "beaking" of the lateral cortex. Incomplete AFFs include only the lateral cortex, whereas complete AFFs involve both cortices and may contain a medial spike. Generalized cortical thickening, prodromal pain, bilateral fractures, and delayed healing are minor features. The exact pathophysiological mechanism of AFFs is still unknown. Alendronate prevents physiological bone resorption by inhibiting osteoclast activity and promoting apoptosis in osteoclasts [2,11]. Therefore, the prolonged use of alendronate may result in oversuppression of bone remodeling, the diminished ability for microfracture repair, and increased bone fragility [2,4].

ASBMR’s most recent epidemiological data indicate that long-term use for more than three years may be linked to an incidence of 100 cases per 100,000 person-years [12]. Lenart et al. claimed that subtrochanteric or shaft fractures were more likely after long-term bisphosphonates, but without excluding the lower possibility of intertrochanteric and femoral neck fractures [9]. The unique features of our case report include three atypical fractures in the same patient, two of which occurred simultaneously and involved all the anatomical areas of the proximal half of the femur (the neck of the femur, the subtrochanteric area, and the proximal diaphysis).

As for the management of bisphosphonate-induced AFFs, this differs according to the type of fracture. For an asymptomatic, incomplete AFF, conservative therapy with limited weight-bearing mobilization may be considered as a treatment option [12]. However, it has been claimed that because the majority of these fractures often progress to complete fractures, nonoperative therapy is not a solid option and that prophylactic fixation is ideal [13]. For a complete AFF and a symptomatic incomplete AFF, a full-length cephalomedullary nail (CMN) or a full-length intramedullary nail (IMN) are the preferred surgical options for stabilizing the fracture and protecting the entire femur [12,14]. For our patient's most efficient and fast mobilization, we determined it would be better to treat both fractures surgically under a single anesthesia.

After an insufficiency fracture, the latest expert-based guidelines suggest the discontinuation of bisphosphonates or any other antiresorptive agent and the alternative administration of calcium and vitamin D supplementation [10,14]. Recently, teriparatide treatment after an AFF has been suggested due to its ability to reverse the oversuppression of bone remodeling but further evaluation and investigation are required [15]. Generally, a drug holiday after five years of continuous use, as well as an annual reevaluation of the medication's necessity, has been recommended in order to prevent insufficiency fractures following long-term bisphosphonate treatment [3,12]. In our case report, we used denosumab after discontinuing bisphosphonates due to our patient's excessive osteoporosis and only after the necessary drug holiday. Concerning our patient, the first AFF happened three years after she started taking alendronate, and the next two occurred simultaneously after 11 years on the same medication. Therefore, the continuation of her alendronate medication for more than eight years after her first right atypical femoral shaft fracture resulted in concurrent bilateral femoral insufficiency fractures. Eventually, this occurrence could further confirm the strong relationship between long-term alendronate treatment and atypical femoral fractures.

## Conclusions

In summary, osteoporosis treatment with long-term alendronate is strongly connected to AFFs. Those could be bilateral, simultaneous, or even presented on different occasions as more than two (three in our patient) covering the proximal femoral areas. The management of AFFs in osteoporotic patients is challenging for orthopaedic surgeons, and when there are implants in situ, that could increase their complexity. The lesson to be learned from our patient is that the doctor should have a high index of suspicion for patients on bisphosphonate treatment who complain of hip or thigh pain and difficulty walking or even standing upright. The longer patients are on bisphosphonate treatment, the more likely it is that they can develop consecutive proximal AFFs, sometimes even with implants in situ.
